# The First Comprehensive Biodiversity Study of Culturable Fungal Communities Inhabiting Cryoconite Holes in the Werenskiold Glacier on Spitsbergen (Svalbard Archipelago, Arctic)

**DOI:** 10.3390/biology11081224

**Published:** 2022-08-16

**Authors:** Justyna Borzęcka, Jakub Suchodolski, Bartłomiej Dudek, Lena Matyaszczyk, Klaudyna Spychała, Rafał Ogórek

**Affiliations:** 1 Department of Mycology and Genetics, University of Wrocław, Przybyszewskiego Street 63-77, 51-148 Wrocław, Poland; 2 Department of Microbiology, University of Wrocław, Przybyszewskiego Street 63-77, 51-148 Wrocław, Poland

**Keywords:** culturable fungi, cryoconite holes, Spitsbergen, Werenskiold Glacier

## Abstract

**Simple Summary:**

Cryoconites are small cavities filled with water on the surface of glaciers in which microorganisms may develop during the thawing period. At the bottom of cryoconite holes, sediment accumulates, consisting of plant and animal debris and inorganic mineral particles. In this study, we provide the first report of fungal communities in cryoconite holes in the Werenskiold Glacier on Spitsbergen (Svalbard Archipelago, Arctic). Overall, we detected 21 species and 2 unassigned species, including micromycetes and macromycetes. Some of the fungi described may be harmful to humans or have biotechnological potential. Most importantly, to the best of our knowledge, we are the first to report the occurrence of *Aspergillus pseudoglaucus*, *Cladosporium allicinum*, *C. ramotenellum*, *Penicillium sumatraense*, *P. velutinum*, *Phanerochaete cumulodentata*, *Bjerkandera adusta*, and *Trametes versicolor* in polar regions.

**Abstract:**

Cryoconite holes on glacier surfaces are a source of cold-adapted microorganisms, but little is known about their fungal inhabitants. Here, we provide the first report of distinctive fungal communities in cryoconite holes in the Werenskiold Glacier on Spitsbergen (Svalbard Archipelago, Arctic). Due to a combination of two incubation temperatures (7 °C and 24 ± 0.5 °C) and two media during isolation (PDA, YPG), as well as classical and molecular identification approaches, we were able to identify 23 different fungi (21 species and 2 unassigned species). Most of the fungi cultured from cryoconite sediment were ascomycetous filamentous micromycetes. However, four representatives of macromycetes were also identified (*Bjerkandera adusta*, *Holwaya mucida*, *Orbiliaceae* sp., and *Trametes versicolor*). Some of the described fungi possess biotechnological potential (*Aspergillus pseudoglaucus*, *A. sydowii*, *Penicillium expansum*, *P. velutinum*, *B. adusta*, and *T. versicolor*), thus, we propose the Arctic region as a source of new strains for industrial applications. In addition, two phytopathogenic representatives were present (*P. sumatraense*, *Botrytis cinerea*), as well as one potentially harmful to humans (*Cladosporium cladosporioides*). To the best of our knowledge, we are the first to report the occurrence of *A. pseudoglaucus*, *C. allicinum*, *C. ramotenellum*, *P. sumatraense*, *P. velutinum*, *P. cumulodentata*, *B. adusta*, and *T. versicolor* in polar regions. In all likelihood, two unassigned fungus species (*Orbiliaceae* and *Dothideomycetes* spp.) might also be newly described in such environments. Additionally, due to experimenting with 10 sampling sites located at different latitudes, we were able to conclude that the number of fungal spores decreases as one moves down the glacier. Considering the prevalence and endangerment of glacial environments worldwide, such findings suggest their potential as reservoirs of fungal diversity, which should not be overlooked.

## 1. Introduction

Cryoconite is a dark granular deposit that accumulates on glacial surfaces. Its structure is complex and consists mainly of an inorganic fraction (mineral fragments such as quartz and silicates), which accounts for up to 95% of total mass, and organic matter (living and dead microorganisms) [1,2]. The spherical mineral particles can be combined with microbial cells (including bacteria, cyanobacteria, archaea, fungi, or algae), forming cryoconite granules. In turn, the mineral dust covering icy surfaces can be a suitable substrate for microorganisms, resulting in the formation of biofilms, which preserve and darken the sediments [1]. As a consequence of its dark color, the heat from solar radiation melts the area covered with cryoconite dust faster than the surrounding surface (due to the lower albedo), creating quasi-circular depressions called cryoconite holes [2,3,4]. They are variously shaped and filled with meltwater containing materials freed from solid particles and melted glacial ice [5]. These depressions are biologically active niches in ice ecosystems due to the unique environmental conditions, which enable the development of a plethora of organisms, including extremophilic microorganisms, providing them with mineral and organic matter to grow upon [6]. It makes them one of the most diverse and species-rich habitats of icy surfaces [7]. Such holes show considerable microbial activity; however, the composition of the microbial community may vary depending on the geographic location, since the characteristics of the local environment may influence the composition of the cryoconites [3,4], as well as the polar season—the hole structure changes throughout the year depending on polar summer and polar winter [8]. For instance, during polar summer, liquid water is found on the glacial surface, enabling microorganisms to sequester nutrients directly from glacier ice, snow cover, and the atmosphere, together with organic and inorganic debris [9], and the process of photosynthesis (algae and cyanobacteria) can provide enough nutrients for the development of a microbial community [5], whereas polar winters bring colder temperatures, making relatively shallow cryoconite holes freeze [10]. In Svalbard, the observed microbial activity in lakes can be even greater, as the water temperature can reach up to 10 °C during the polar summer, and additional nutrients are provided either from seabirds and mammalian droppings or from rock weathering [9].

The specific cryoconite microflora is very diverse, and some of the obtained microorganisms are mutual for the several studied regions, although some of them are considered to be distinctive for a given area. This is understandable because, depending on the location, the glacier surface and cryoconite holes may exhibit different conditions, such as oxygen saturation, nutrient abundance, or light availability [11]. Due to harsh conditions, fungi are isolated from those areas less frequently and belong mainly to psychrophilic and psychrotolerant species. Studies show that genera such as *Rhodotorula* (e.g., *Rhodotorula psychrophenolica*, *R. svalbardensis*, or *R. glacialis*) [3,12], *Cryptococcus*, *Debaryomyces*, *Torulopsis*, and *Candida* [13]; the Pezizales order, represented by the genera *Choiromyces* (specifically *Choiromyces meandriformis*), *Hydnotrya* (*Hydnotrya tulasnei*), or *Verpa* (*Verpa bohemica*) [5]; and Pezizales-related *Dothideomycetes* (e.g., *Alternaria* sp.) [12] or *Thelebolus* are, among others, far isolated from such areas. The latter species is associated with guano, and some studies suggest a potential avian impact on cryoconite [3]. Additionally, other species obtained in previous research were composed of micromycetes—such as *Penicillium* spp., *Phialophora* sp., *Cladosporium* spp., *Circinella* sp., [12], Chytridiomycota, or the Ascomycota group (*Acremonium*, *Articulospora*, *Ascochyta*, *Preussia*, *Pseudeurotium*, *Varicosporium* genera), which are related to plant endophytes (and in all probability, many of them were transported by wind to the cryoconite holes from plant litter or soil) and, finally, basidiomycetous yeast, such as *Mrakia* sp. (e.g., *M. robertii*), *Varicosporium elodeae*, or *Glaciozyma watsonii*—isolated from glacial habitats, predominantly from meltwater [3,6,12,14]. It is presumed that filamentous fungi previously isolated from cryoconite holes may be significant decomposers and constitute a crucial element of such saprophytic communities [3].

Fungi are pivotal for the cycling of carbon and nutrients (including N) in the terrestrial ecosystems of the Arctic [15,16]. However, microorganisms in cryoconite holes are exposed to multiple stresses that result from fluctuations in environmental conditions, as well as low temperatures. These fluctuations include freeze–thaw cycles, high pH and low nutrient availability, decreased diffusion rate, increased fluid viscosity, osmotic stress, and exposure to UV radiation [11]. Thus, the ability of psychrophilic and psychrotolerant microorganisms to survive and grow in icy and inhospitable environments is the result of many adaptation strategies. To survive in harsh environments, fungal strains have developed a series of living strategies and function as saprobes, symbionts, plant and animal parasites, and pathogens to perform various ecological roles [17], e.g., the majority of Arctic plants’ nutrient uptake is accomplished by mycorrhizal symbioses, and the proportion of Arctic vegetation biomass associated with mycorrhizal fungi has been estimated to range from 17% to 100% [18]. The most frequently observed adaptations of functional fungi to cold environments that include changes are ones in the phospholipid bilayer composition, structural modifications of enzymes, the production of cold shock proteins, and ice-nucleating proteins anchored in the outer membrane and capable of ice nucleating by binding water molecules in a specific conformation [15,16,19,20]. Moreover, psychrotolerant species are usually the microorganisms most frequently found in cold environments, perhaps because they have better nutritional adaptability or due to horizontal gene transfer from mesophiles [21,22]. As might be expected, such microorganisms are capable of fulfilling multiple and diverse roles in such cold and harsh environments. They can enhance the quality of the soil structure, fix nitrogen, or break down organic matter for plants to absorb or deteriorate pollutants [23].

The aim of this study was to be the first comprehensive analysis of the fungal composition of cryoconites from the Werenskiold Glacier located in Spitzbergen on the Svalbard Archipelago (Arctic). The climate of Svalbard is quite varied and seasonally dependent, with high temperature variations throughout the year and a low amount of precipitation. Such variable environmental conditions, as well as animal migrations, influence the local microflora occurring in waters, soil, and glaciers. Thus, studying the mycobiota composition of this Arctic area might facilitate analyzing the relationship between the diversity of microbial species and the climatic features of the region.

## 2. Materials and Methods

**Figure 1:**
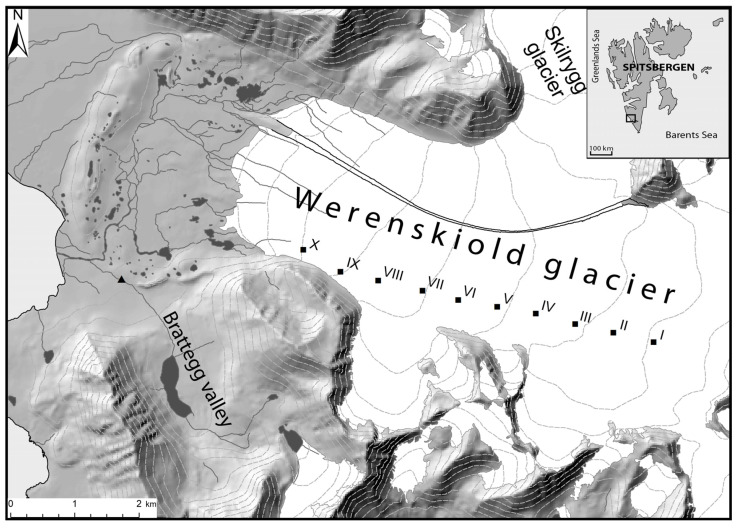
Geographic location of the Werenskiold Glacier on Spitsbergen (Svalbard Archipelago, Arctic) and study sites from I to X.

The study was conducted on the basis of the authorization granted by the Svalbard Governor and permission for project RIS-ID 10604, Microbiological Diversity of the Arctic Biosystems. Samples were taken on 2 August 2016, during Arctic summer, from cryoconite sediments on the Werenskiold Glacier in Spitzbergen, on the Svalbard Archipelago (Figure 1), from 10 locations: (I) (N 77°03.827′, E 15°26.374′), (II) (N 77°03.880′, E 15°25.232′), (III) (N 77°03.950′, E 15°24.192′), (IV) (N 77°03.997′, E 15°22.898′), (V) (N 77°04.039′, E 15° 21.857′), (VI) (N 77°04.074′, E 15°20.739′), (VII) (N 77°04.178′, E 15°19.674′), (VIII) (N 77°04.220′, E 15°18.201′), (IX) (N 77°04.295′, E 15°17.262′), and (X) (N 77°04.450′, E 15°16.045′). Sediment samples were placed aseptically in individually wrapped and sterile conical polypropylene test tubes (50 mL) with screw caps (Biologix, China), with each sample in a separate tube. Samples were stored at −20 °C until the microbiological analyses.

### 2.1. Study Area

Samples were collected in Spitsbergen, which is the largest island of the Svalbard Archipelago in the Arctic Ocean (latitude between 74° and 81° N). The specific climate results from its location between the cold Arctic East Spitsbergen Current and the Atlantic West Spitsbergen Current. The latter alters and moderates the temperature in Spitsbergen, making winter temperatures up to 20 °C higher than in other Arctic regions. Spitsbergen fauna and flora are affected by the occurrence of polar day and night cycles, as well as windy and cold winters, short summers, and a small quantity of precipitation. Due to the region’s vulnerability to climatic changes, environmental conditions are harsh and inhospitable; therefore, the species that inhabit them are well adapted. The flora of Svalbard consists of about 170 species of vascular plants (including three endemic species: Svalbard quinquefoil (*Potentilla × insularis*), Svalbard saxifrage (*Saxifraga svalbardensis*), and Svalbard saltmarsh grass (*Puccinellia svalbardensis*), which are accompanied by mosses (nonvascular plants) as well as fungi, algae, and lichens, while the animals are represented by numerous bird species. Mammals, such as the Arctic fox, the Svalbard reindeer, polar bears, seals, and sea lions, are the native species inhabiting Svalbard. All of the above climate features influence the composition of Svalbard microflora [23,24,25,26].

### 2.2. Mycological Analysis of Samples

Prior to testing, samples were thawed, and 3 g of cryoconite sediment was placed in individually wrapped and sterile conical polypropylene test tubes (25 mL) with screw caps (FL Medical, Italy) with 12 mL physiological salt solution (0.85% NaCl), shaken at room temperature (20 min; 25 ± 1 °C). Then, the samples were diluted 25×, 50×, 500×, and 5000×; vortexed; spread on plates in triplicates; and incubated for 5–56 days at 7 °C and 24 ± 0.5 °C on PDA (potato dextrose agar, BioMaxima, Lublin, Poland) and YPG (yeast extract peptone glucose: 10.0 g·L^−1^ yeast extract, 20.0 g·L^−1^ peptone, 20.0 g·L^−1^ glucose, 15.0 g·L^−1^ agar). Pure cultures were obtained with the single spore method on PDA medium and were subcultured on PDA slants for morphological and molecular identification. At this point, fungal colony-forming units (CFUs) per 1 g of cryoconite sediment were calculated.

### 2.3. Fungal Identification

A combination of phenotypic and genotypic methods was used for fungal identification. Pure cultures were analyzed with both micro- and macroscopic observations. Preliminary phenotypic identification was performed on PDA, Czapek yeast autolysate agar (CYA, 30.0 g·L^−1^ sucrose, 15 g·L^−1^ agar, 5.0 g·L^−1^ yeast extract, 3.0 g·L^−1^ NaNO_3_, 1.0 g·L^−1^ K_2_HPO_4_, 0.5 g·L^−1^ KCl, 0.5 g·L^−1^ MgSO_4_·7H_2_O, 0.01 g·L^−1^ FeSO_4_·7H_2_O), Czapek–Dox agar (1.2% agar, BioMaxima, Poland), and malt extract agar (MEA, BioMaxima, Poland) in the case of *Aspergillus* and *Penicillium* spp. The observed features included colony color and growth, as well as the occurrence of specific morphological structures such as spores. The isolates were analyzed according to diagnostic keys and monographs [27,28,29,30,31,32,33,34,35,36,37,38,39,40,41,42,43,44].

To confirm species affiliation, the fungal rDNA ITS (internal transcribed spacer) was sequenced. DNA was isolated from fungal colonies cultured on PDA using the Bead-Beat Micro AX Gravity (A&A Biotechnology, Gdańsk, Polska) according to included protocol. Fungal rDNA ITS was amplified using the primer ITS1 (5′-TCCGTAGGTGAACCTGCGG-3′) and ITS4 (5′-TCCTCCGCTTATTGATATGC-3′) [45]. PCR was performed in a T100 Thermal Cycler (Bio-Rad), according to Ogórek et al. [46]. The PCR products were verified by electrophoretic separation on a 1.2% agarose gel, and, subsequently, they were purified using Clean-UP (A&A Biotechnology) and sequenced by Macrogen Europe (Amsterdam, The Netherlands, http://dna.macrogen.com/eng/, accessed on 25 April 2021).

### 2.4. Data Analyses

The raw fungal sequence reads were analyzed using the BioEdit Sequence Alignment Editor (http://www.mbio.ncsu.edu/bioedit/bioedit.html, accessed on 10 May 2021) and compared with those deposited in the GenBank of the National Center for Biotechnology Information (NCBI, Bethesda, MD, USA) using the BLAST algorithm (http://www.ncbi.nlm.nih.gov/, accessed on 10 May 2021). Generated rDNA ITS fungal sequences were submitted to NCBI GenBank under accession numbers from MZ045861 to MZ045883. To determine the diversity of fungal communities at specific research sites, the Shannon Diversity Index (H) was used and calculated with the following equation: H = −∑Pi(lnPi), where Pi stands for the proportion of each community in the sample [47,48].

## 3. Results

All fungi isolated in the study at the 10 sampling points were clustered into 23 major groups based on macro- and micromorphology. Next, the rDNA ITS sequencing of the representatives of the groups was carried out; they were given numbers from UWR_219 to UWR_241, which resulted in affiliating the fungi into 23 different species (Table 1). The lengths of the PCR products of the sequences ranged from 363 to 525 bps. All sequences were submitted to GenBank under accession numbers from MZ045861 to MZ045883. Based on the BLAST analysis, the E values were zero, the percentages of the query cover amounted to 100%, and the identity ranged from 97.62% to 100%. The identified Arctic fungi belong to either Ascomycota (19 isolates) or Basidiomycota (four isolates) (Table 1).

**Figure 2:**
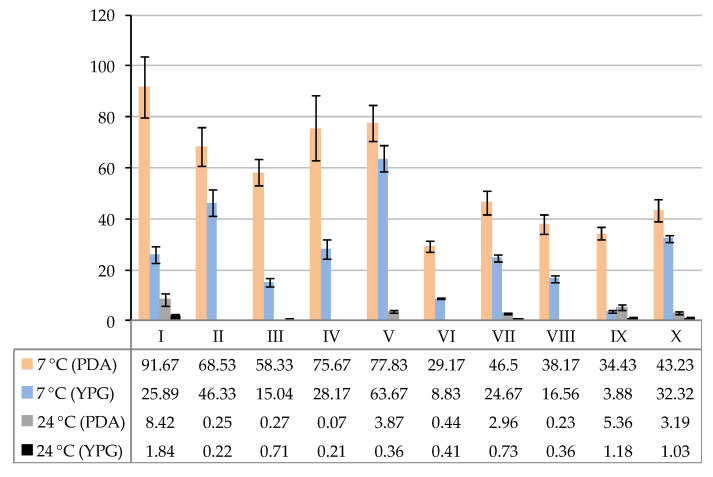
The number (CFU × 10^2^ per 1 g ± SD) of fungi cultured from the cryoconite holes in the Werenskiold Glacier (Spitsbergen) and incubated at 7 or 24 °C on PDA and YPG media: I–X—study sites.

Mycological investigations in the study were performed on two culture media (PDA and YPG) and at two incubation temperatures (7 and 24 ± 0.5 °C). The best medium in terms of obtaining the highest number of fungal CFUs per 1 g of sediment was PDA at both incubation temperatures. On the other hand, the amount of psychrophilic and psychrotolerant fungi growing at 7 °C was higher than those grown at 24 °C (potentially mesophilic fungi) in both media (Figure 2). However, at a higher incubation temperature, a greater number of species was obtained (10 species at 7 °C on PDA and YPG; 17 species at 24 °C on PDA; 13 species at 24 °C on YPG) (Table A1 and Table A2).

The CFU values of fungi capable of growing at 5 °C were from 38.17 to 91.67 × 10^2^ per 1 g of sediment in the case of PDA, and from 3.88 to 63.67 91.67 × 10^2^ in the case of YPG. The highest values of fungal CFUs were recorded in study sites I, IV, and V on PDA and in study site V on YPG. In turn, the concentration of fungi grown at 24 °C ranged from 0.07 to 8.42 CFU × 10^2^ per 1 g of sediment and from 0.22 to 1.84 CFU × 10^2^ on PDA and YPG, respectively. The highest values of CFU for fungi capable of growing at 24 °C were detected at the sampling site I on PDA, and, in the case of the YPG, the values were at the same level (Figure 2).

**Figure 3:**
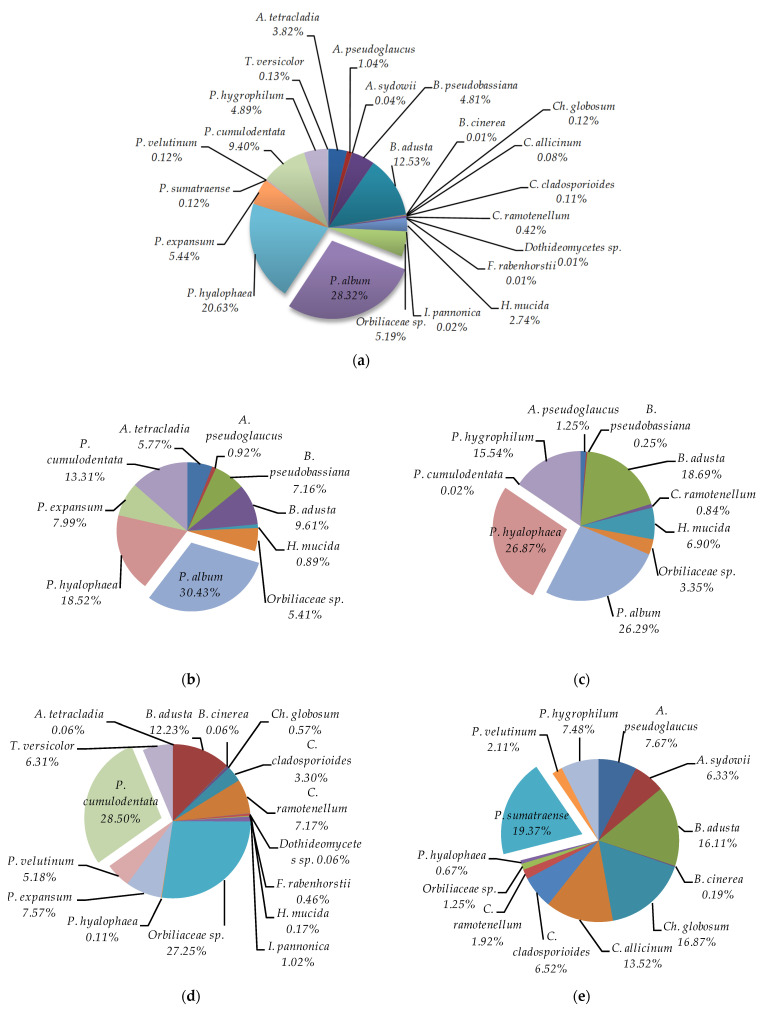
Percentage of each fungus contributing to the total fungi cultured from the cryoconite holes in the Werenskiold Glacier (Spitsbergen) using different culture media and different incubation temperatures: (**a**) overall for all incubation temperatures and for all culture media, (**b**) on PDA incubated at 7 °C, (**c**) on YPG incubated at 7 °C, (**d**) on PDA incubated at 24 °C, and (**e**) on YPG incubated at 24 °C.

Overall, the most frequently isolated species in the study was *Parengyodontium album*, which accounted for 28.32% of all fungi (23 species) grown across all experimental variants (Figure 3a). This species was also the most often isolated on PDA and incubated at 7 °C, and it contributed 30.43% of all cultured fungi—10 species (Figure 3b). In turn, *Patinella hyalophaea* was most abundant on YPG at 7 °C (26.87% of all fungi—23 species), *Phanerochaete cumulodentata* on PDA incubated at 24 °C (28.50% of all fungi—16 species), and *Penicillium sumatraense* on YPG incubated at 24 °C (19.37% of all fungi—13 species) (Figure 3c–e).

**Figure 4:**
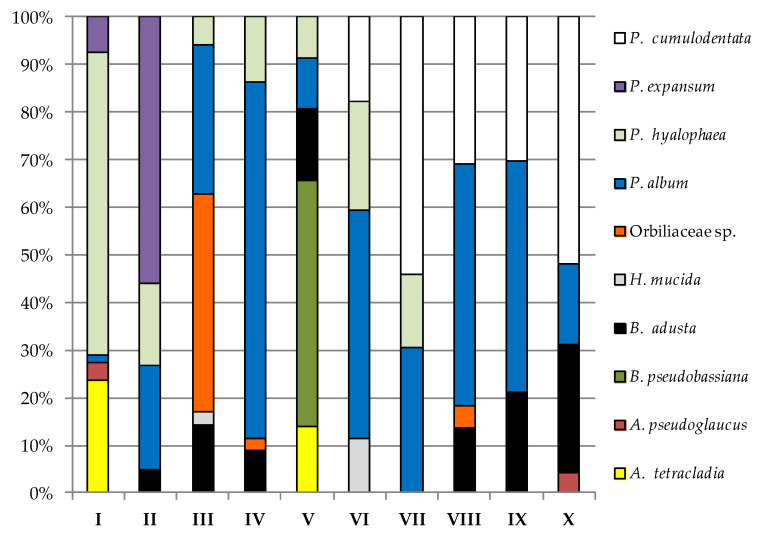
Percentage of each fungus contributing to the total fungi cultured on PDA from the cryoconite holes in the Werenskiold Glacier (Spitsbergen) at 7 °C from study sites I to X.

**Figure 5:**
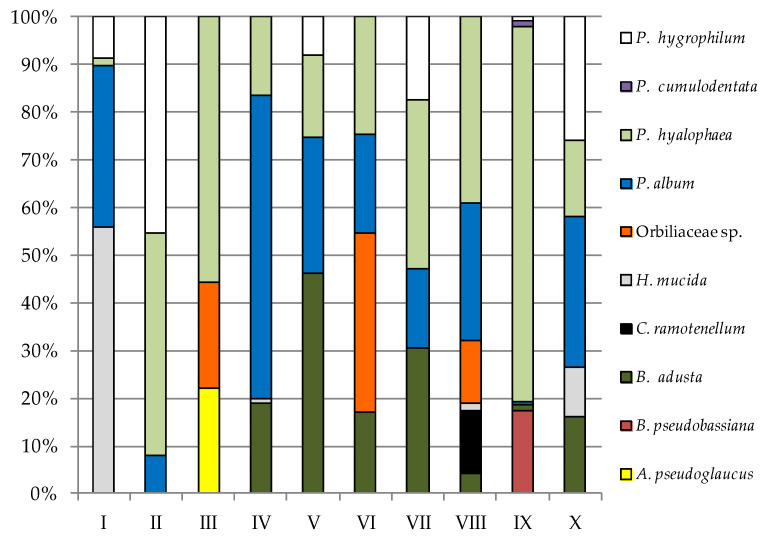
Percentage of each fungus contributing to the total fungi cultured on YPG from the cryoconite holes in the Werenskiold Glacier (Spitsbergen) at 7 °C from study sites I to X.

**Figure 6:**
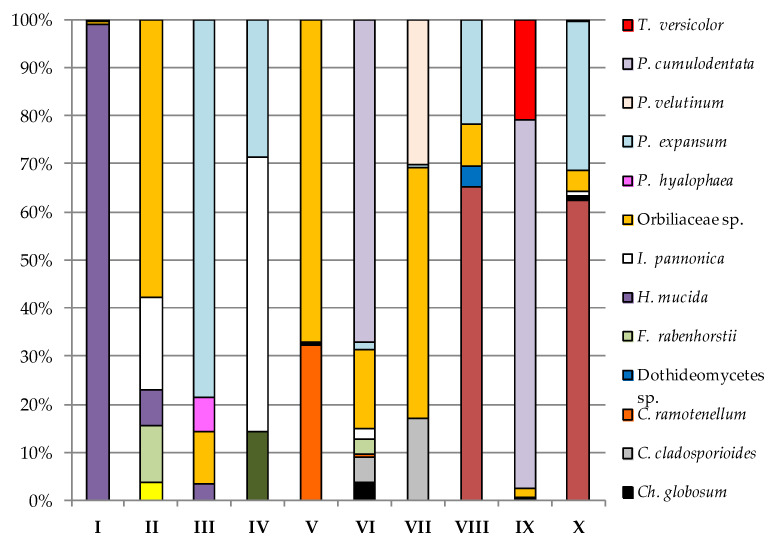
Percentage of each fungus contributing to the total fungi cultured on PDA from the cryoconite holes in the Werenskiold Glacier (Spitsbergen) at 24 °C from study sites I to X.

**Figure 7:**
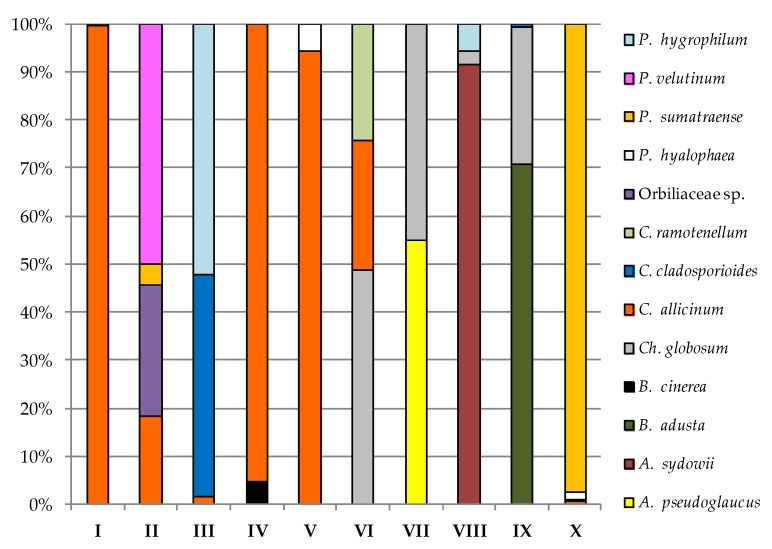
Percentage of each fungus contributing to the total fungi cultured on YPG from the cryoconite holes in the Werenskiold Glacier (Spitsbergen) at 24 °C from study sites I to X.

Particular study sites differed qualitatively and quantitatively in terms of fungi at a given incubation temperature on a given medium and between them (Figure 4, Figure 5, Figure 6 and Figure 7, Table A1 and Table A2). The most frequently isolated species in study site I in the cultivation conditions typical for psychrophilic and psychrotolerant fungi on PDA was *P. hyalophea*, and for YPG, it was *Holwaya mucida*; they accounted for 63.64% and 55.8% of all isolated fungi, respectively (Figure 4 and Figure 5). *P. hyalophea* was also the dominant species in the case of YPG in five study locations (II, III, VII, VIII, and IX) and constituted 35.14% to 78.61% of all isolated fungi (Figure 5). In turn, *Penicillium expansum* was the most frequently cultured species on PDA in study site II (55.93%), and *Orbiliaceae* sp. was the most frequently cultured species in study site III on the same medium (45.72%), as well as in the study site VI on YPG (37.71%). Sampling sites IV, VI, VIII, and IX on PDA and IV and X on YPG were most often populated by *P. album*, which constituted from 31.62% to 74.89% of all isolated fungi. On the other hand, *P. cumulodentata* was the most frequently cultured species on PDA from study sites VII (54.12%) and X (51.82%), while study site V was dominated by *Beauveria pseudobassiana* and *Bjerkandera adusta* on PDA (51.82%) and YPG (46.07%, respectively (Figure 4 and Figure 5).

In the case of sampling site I on PDA and YPG, the dominance of *H. mucida* and *Cladosporium allicinum* was noted, respectively, to be above 99% of all isolated fungi, taking into account the higher incubation temperature of the biological material (Figure 6 and Figure 7).

*C. allicinum* was also the most abundant species isolated from sites IV and V with the use of YPG at a level of about 95% (Figure 7). In turn, *Orbiliaceae* sp. taken from locations such as II (57.69%), V (67.18%), and VII (51.85%) most often inhabited PDA. Study site II samples on PDA were most populated by *P. expansum* (78.57%), and location IV samples on the same medium were dominated by *Itersonilia pannonica* (57.14%) (Figure 6). In the case of sampling sites VI (67.16%) and IX (76.82%), on PDA, *P. cumulodentata* was the most isolated species, and *Cladosporium ramotenellum* was the dominant species from study sites VIII (65.22%) and X (62.31%) on the same medium (Figure 6). On the other hand, the most abundant species on YPG was *Penicillium velutinum* from study site II (50%), *Pseudeurotium hygrophilum* from study site III (52.11%), *Chaetomium globosum* from study site VI (48.78%), *Aspergillus pseudoglaucus* from study site VII (54.79%), *Aspergillus sydowii* from study site VIII (91.67%), *B. adusta* from study site IX (70.59%), and *P. sumatraense* from study site X (97.56%) (Figure 7).

**Figure 8:**
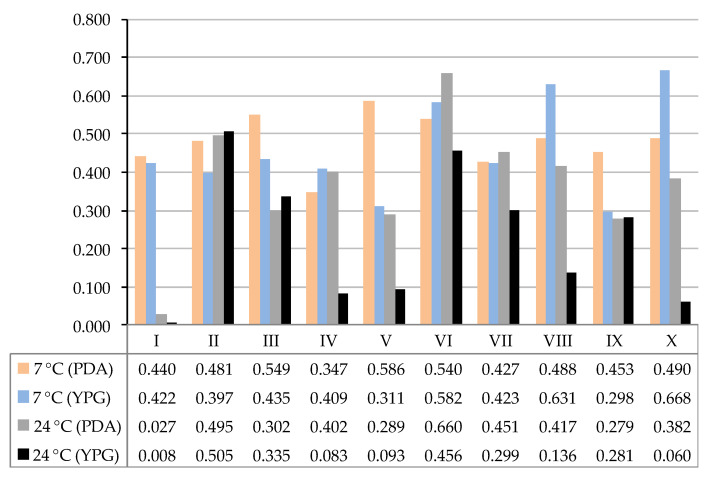
The values of the Shannon Diversity Index calculated to determine the diversity of fungal communities from specific research sites: I–X—study sites.

The study sites differed from each other in terms of the diversity of the fungal species, which is also illustrated by the Shannon Diversity Index values (Figure 8). Overall, the incubation of the research material at 7 °C allowed us to obtain greater fungus species biodiversity. The mean values of the Shannon Diversity Index for all research locations were 0.4801, 0.4576, 0.3704, and 0.2256 for the following variants of the experiment: 7 °C (PDA), 7 °C (YPG), 24 °C (PDA), and 24 °C (YPG), respectively. The greatest species diversity of fungi was recorded for study site X on PDA incubated at 7 °C (0.668), and the lowest was for study site I on YPG incubated at 24 °C (0.008) (Figure 8).

## 4. Discussion

Although the microbial populations of the surface ice and cryoconite holes of the Werenskiold Glacier have been studied in the past [49], a mycological investigation has not been carried out so far. The Arctic area is one of the most challenging natural habitats for life. However, a wide diversity of microorganisms, both prokaryotes and eukaryotes, is constantly being reported to thrive in such areas. Every step taken in gaining a deeper understanding of the microbial biodiversity in such unique environments contributes to our recognition of the composition and dynamics of microbial communities and their roles in Arctic ecology. In particular, since profound changes are most likely to occur in patterns of vegetation and the size of soil carbon pools in the Arctic by the end of this century [15], it is necessary to know more about which types of species of “decomposer” fungi are present and to try to define their potentially pivotal roles in these ecosystems.

In general, the viable cell count made in the present study showed that the cryoconite holes at higher latitudes support higher fungal diversity. This was especially true when using PDA medium at 4 °C. Thus, it can be concluded that the number of fungal spores decreases as one moves down the glacier. This is partially in agreement with mycological studies on cryoconite holes in the Midre Lovénbreen Glacier [14]. Singh and Singh [14] suggested that the most likely reason is lower temperatures at higher altitudes.

The combination of classical and molecular identification approaches showed the presence of 23 different fungal species from Werenskiold Glacier cryoconite sediments. Species of *P. album* and *P. hyalophaea* were, overall, the most abundant, as well as among the fungi cultured at 7 °C. The extremophilic *P. album* species has been detected in polar/Arctic areas before [50]. However, its taxonomic position has been changed quite frequently, and it has been regularly reported under its former taxonomic name, *Engyodontium album*. Interestingly, this fungus is generally considered to be a mesophile [51] despite the fact that it is capable of surviving at subzero temperatures, as proved by numerous studies conducted in Arctic or Antarctic environments [52,53]. Additionally, *P. album* has been detected in the stratosphere, which is not only characterized by subzero temperatures but also high UV exposure [54]. Furthermore, *P. album* has frequently been reported in: cultural heritage locations (historical buildings, museums, libraries, and touristic sites) [50]; caves, where humidity levels can approach 100% [55]; and marine environments (sediments, sponges, and directly from seawater) [56,57,58,59,60]. In conclusion, the detection of *P. album* around the world, including in extreme environments, shows its ubiquitous nature and explains its high abundance in this study. On the other hand, there are very few reports on *P. hyalophaea*, the other most abundant species isolated herein. Most likely, a report on *P. hyalophaea* in New Brunswick, Canada, in 2013 was the first since its original discovery in 1875 [61]. Baral and Carter [61] suggested an association between *P. hyalophaea* and semiaquatic habitats. This is in agreement with the recent report of *P. hyalophaea* from a torrent watercourse in a bog complex of an altimontane karst polje in the Dinaric Alps [62]. In the case of polar regions, *P. hyalophaea* was recently isolated from wood samples from Deception Island (Antarctica) [63] and Western Greenland [64]. Additionally, on King George Island (Antarctica), the fungus was reported in lacustrine sediment cores collected from a lake [65].

*P. album* and *P. hyalophaea* were the most frequently isolated species overall; however, when taking into account only fungi isolated at 24 °C, *P. cumulodentata* and *P. sumatraense* were the most abundant on PDA and YPG media, respectively. It can be concluded that the effectiveness of a successful, culture-based mycological analysis depends, among other things, on combining diverse conditions, such as different temperatures and types of media [66]. Nevertheless, only a few reports regard *P. cumulodentata*, including its original description in 1976 [67]. Later, the species was also isolated in Finland, Lithuania, and Ukraine [36]. Most recently, *P. cumulodentata* was reported in Russia, in the following regions: Arkhangelsk Oblast, Plesetsky District, Kenozersky National Park, and Shishkino Village [68]. Similarly, only a few reports regard *P. sumatraense*, which was described i.a. in Tunisia [69] and the Philippines [70]. In both cases, the fungus was described as a phytopathogen infecting apples or bamboo. Most recently, *P. sumatraense* was isolated using an algal trap and described as being capable of assimilating *Chlorella vulgaris* [71]. Due to a limited number of reports on both fungi, we conclude that we are the first to describe both species (*P. cumulodentata* and *P. sumatraense*) in polar regions. Further studies on those still unknown fungal species might explain their origin in the Arctic.

However, *P. sumatraense* was not the only Aspergillaceae family representative isolated herein. These also included: *A. pseudoglaucus*, *A. sydowii*, *P. expansum*, and *P. velutinum*. *A. sydowii* and *P. expansum* were already reported in polar regions [72,73]. All four species are well known for their wide metabolic abilities and are constantly reported as being useful in industry and biotechnology. Some notable examples are: the fermentation of katsuobushi into karebushi (traditional Japanese cuisine) by *A. pseudoglaucus* [74]; the production of volatile compounds for traditional Chinese dry sausages by *A. pseudoglaucus* [75]; the production of mycophenolic acid by *A. pseudoglaucus* [76]; bioremediation by *A. sydowii* [77,78,79]; the production of monoterpenoids by *A. sydowii* [80]; the production of different polyketides by *A. sydowii* and *P. velutinum* [80,81,82]; the production of xylanases by *A. sydowii* [83]; the production of fructooligosaccharides by *P. expansum* [84]; the production of patulin, citrinin, and lipases by *P. expansum* [85,86,87]; and the production of pectin lyase and protease by *P. velutinum* [88]. The isolation and characterization of novel environmental strains of microorganisms that are already known for their biotransformation abilities may lead to the further improvement and better understanding of biotechnological processes, especially in the case of extremophilic strains (such as those in the Arctic), which might possess cold-adapted variants (protein muteins) of already known enzymes. In addition, some already known species, new strains of which have been isolated from extreme environments, might possess yet undiscovered traits. A notable example is the isolation of *Pseudomonas fluorescens* BD5 from Svalbard [89] and the identification of its novel biosurfactant metabolite, pseudofactin II [89]. Pseudofactin II was later investigated for its antiadhesive, antitumor, and general biotechnological properties [90,91,92,93,94]. Thus, the potential usefulness of our strains has yet to be investigated.

On the other hand, the Cladosporiaceae family is mostly associated with seasonal allergies. *C. cladosporioides* rarely causes infections in humans and plants; however, its spores might trigger severe asthmatic reactions [95,96]. A few years before the present study, *C. cladosporioides* was reported in the Austre Brøggerbreen deglaciation area, Ny-Alesund, Svalbard [97]. However, the other *Cladosporium* spp. Representatives, *C. allicinum* and *C. ramotenellum*, are most likely to have been first described as inhabiting polar regions in the present study. *C. allicinum* has been frequently isolated from indoor air samples in Europe [98,99], but it has also been described as inhabiting the dust on the skull of the blue whale skeleton at the Natural History Museum, London [100]. Contrarily, *C. ramotenellum* is mostly associated with plants but presents a dual nature. On one hand, the species has been described as a beneficial mycorrhizal fungus [101,102], but it has also as been the cause of ripening in grape berries and mandarins [103,104]. The presence of *C. allicinum* and *C. ramotenellum* might be associated with migrating birds, which arrive for the breeding period on the nearby coast (Wedel Jarlsberg Land) [105].

To our surprise, we were able to isolate a few representatives of macromycetes, which included: *B. adusta, H. mucida, Orbiliaceae* spp., and *T. versicolor*, out of which, only *H. mucida* has been previously described in polar areas (Palmer Archipelago on the Antarctic Peninsula and Ross Island, Antarctica) [106]. *B. adusta* and *T. versicolor* are both white-rot fungi (WRF) capable of degrading naturally occurring lignin due to the production of ligninolytic extracellular oxidative enzymes. However, WRF usually secrete a variety of other extracellular enzymes during their secondary metabolism, triggered by nutrient exhaustion. The non-specificity of such enzymes enables them to transform a great variety of recalcitrant and hazardous pollutants [107]. Thus, both species have been constantly reported for their bioremediation capabilities, for example, the biodegradation of i.a. dyes, including textile dyes [108,109,110,111,112,113,114,115,116,117]; hexachlorocyclohexane (HCH) [118]; pesticides [119]; pentachlorophenol (PCP) [120]; pharmaceutical pollutants (e.g., ibuprofen) [121]; polycyclic aromatic hydrocarbons (PAH) [122]; phenanthrene [123]; and the biosorption of heavy metal ions [124]. The last identified macromycetes are a representative of the *Orbiliaceae* family. *Orbiliaceae* includes several genera, such as *Arthrobotrys*, *Dactylella*, *Dactylellina*, *Monacrosporium*, and *Orbilia* [125,126]. It is interesting that *Orbiliaceae* members represent the majority of predatory, nematode-trapping fungi; however, their taxonomic classification is ongoing [127,128,129]. It is most likely for this reason that we were unable to assign the UWR_233 isolate to specific species. However, the present isolate displayed a 100% identification with the reported *Orbiliaceae* sp. 1 MK-2015, which was retrieved from extremely acidic (pH < 3) soil samples collected from the Czech Republic [130]. Thus, we hypothesize that UWR_233 might possess some unique abilities, which explains its Arctic origin. Similarly, the micromycete *Dothideomycetes* sp. UWR_229 isolate could not be assigned to a specific species due to the complex taxonomic history of the *Dothideomycetes* class [131,132]. However, *Dothideomycetes* are mostly associated with freshwater habitats [133,134], which might explain their origin in cryoconite holes.

However, we were also able to isolate a few micromycetes already associated with polar habitats. *A. tetracladia* is a ubiquitous aquatic fungus, previously isolated from cryoconite in Midre Lovénbreen Glacier [80]. Entomopathogenic *B. pseudobassiana* has been isolated from Zackenberg, Danmarkshavn, and the Ritenbenk region of Greenland [135]. *Ch. globosum* has been reported in samples from cryopegs and boreholes located in the tundra zone of the Kolyma lowland near the East Siberian Sea [136]. *F. rabenhorstii* is an ectomycorrhizal fungus (usually found on the root tips of *Pinus sylvestris*), which has been previously isolated within the Dryas octopetala zone of Svalbard, close to Ny-Ålesund in the High Arctic [137]. Previously, it has been estimated that mycorrhizal fungi supply Arctic plants with ~61–86% of the host plants’ nitrogen [138]. Identifying *F. rabenhorstii* spores in cryoconite holes implies that mycorrhiza is particularly beneficial in arctic ecosystems where low nutrient availability occurs. Bjorbækmo et al. [138] suggested that, despite low plant diversity in Arctic ecosystems, there is high diversity in the root-associated fungal communities. Another species, *I. pannonica,* is a cold-adapted yeast commonly found in polar regions, i.a. isolated from glacial meltwater and ice from the Patagonian Andes (Argentina) under its previous name, *Udeniomyces pannonicus* [139]. *P. hygrophilum* was isolated from a lake sediment core obtained from the Trinity Peninsula, Hope Bay, northeastern Antarctic Peninsula [140]. Additionally, previously, we were the first to describe *B. cinerea* in the Arctic [105]. The species, commonly known as a “gray mold”, is a phytopathogen most notable for infecting wine grapes [105].

In relation to Svalbard and cryoconite holes, using the proposed culture-dependent and independent techniques, it should be possible in future studies to test whether the acquired species are stagnant or if new fungal species will enter the region. Additionally, future studies are necessary in order to facilitate cross-comparisons between current and future results.

## 5. Conclusions

Our study contributes to gaining new knowledge about the diversity of cold-adapted fungi inhabiting cryoconite holes in the Werenskiold Glacier on Spitsbergen (Svalbard Archipelago, Arctic). Overall, we isolated 23 different fungi (21 species and 2 unassigned to species). Four were representatives of macromycetes. One of the most commonly isolated species was *P. album*. To the best of our knowledge, our research has allowed for the first detection of some fungal species in Arctic ecosystems (*A. pseudoglaucus, C. allicinum*, *C. ramotenellum*, *P. sumatraense*, *P. velutinum*, *P. cumulodentata*, *B. adusta*, and *T. versicolor*). Not only have we provided new insight into the biology of these species, but we can also report that some of them may present a threat to local plants (*P. sumatraense*, *B. cinerea*) and immunocompromised patients and animals (*C. cladosporioides*). Furthermore, we believe that polar sites may be a source of new strains for biotechnological applications, some examples being *A. pseudoglaucus*, *A. sydowii*, *P. expansum*, *P. velutinum*, *B. adusta*, and *T. versicolor*. We also showed that the number of fungal spores decreases as one moves down the glacier. Therefore, the species biodiversity of this group of fungi in polar ecosystems most likely depends on the temperature and the abundance of local wildlife (animals and plants) living in this type of site.

## Figures and Tables

**Table 1:** Fungi cultured from cryoconite holes in the Werenskiold Glacier (Spitsbergen) on different media and at incubation temperatures. The BLAST analysis was performed on 25 April 2021 (all values of query cover were 100%; all E values were zero).

Fungi Isolated from Glacier Samples					Identity with Sequence from GenBank	
Isolate Number	Identified Fungi	Phylum	GenBank Accession No.	The Sequence Length (bp)	Identity, %	Accession
UWR_219	*Articulospora tetracladia*	*Ascomycota*	MZ045861	409	99.27	LC131004.1
UWR_220	*Aspergillus pseudoglaucus*	*Ascomycota*	MZ045862	488	100.00	MH630012.1
UWR_221	*Aspergillus sydowii*	*Ascomycota*	MZ045863	513	100.00	MN809362.1
UWR_222	*Beauveria pseudobassiana*	*Ascomycota*	MZ045864	502	100.00	MT241786.1
UWR_223	*Bjerkandera adusta*	*Basidiomycota*	MZ045865	481	100.00	MT133795.1
UWR_224	*Botrytis cinerea*	*Ascomycota*	MZ045866	439	100.00	KP900730.1
UWR_225	*Chaetomium globosum*	*Ascomycota*	MZ045867	443	100.00	MN453401.1
UWR_226	*Cladosporium allicinum*	*Ascomycota*	MZ045868	506	100.00	MK460808.1
UWR_227	*Cladosporium cladosporioides*	*Ascomycota*	MZ045869	498	100.00	MK761055.1
UWR_228	*Cladosporium ramotenellum*	*Ascomycota*	MZ045870	452	100.00	N636231.1
UWR_229	*Dothideomycetes* sp.	*Ascomycota*	MZ045871	377	97.62	KJ508303.1
UWR_230	*Fimetariella rabenhorstii*	*Ascomycota*	MZ045872	406	100.00	MN984305.1
UWR_231	*Holwaya mucida*	*Ascomycota*	MZ045873	388	98.46	MN749367.1
UWR_232	*Itersonilia pannonica*	*Basidiomycota*	MZ045874	525	100.00	KX067837.1
UWR_233	*Orbiliaceae* sp.	*Ascomycota*	MZ045875	490	100.00	LN901113.1
UWR_234	*Parengyodontium album*	*Ascomycota*	MZ045876	492	100.00	MT279507.1
UWR_235	*Patinella hyalophaea*	*Ascomycota*	MZ045877	400	99.75	MN833368.1
UWR_236	*Penicillium expansum*	*Ascomycota*	MZ045878	489	100.00	MT218335.1
UWR_237	*Penicillium sumatraense*	*Ascomycota*	MZ045879	465	99.79	MH971259.1
UWR_238	*Penicillium velutinum*	*Ascomycota*	MZ045880	515	100.00	AF033448.1
UWR_239	*Phanerochaete cumulodentata*	*Basidiomycota*	MZ045881	432	99.77	MH971273.1
UWR_240	*Pseudeurotium hygrophilum*	*Ascomycota*	MZ045882	448	100.00	MF375774.1
UWR_241	*Trametes versicolor*	*Basidiomycota*	MZ045883	363	100.00	MT000476.1

## Data Availability

Not applicable.

## Appendix A

**Table A1:** Fungi cultured from cryoconite holes (CFU × 10^2^ per 1 g) in the Werenskiold Glacier (Spitsbergen) at 7 °C on different media: ^1^ I–X—study sites; ^2^ “—” means not detected.

Fungi	Study Sites																			
	I ^1^		II		III		IV		V		VI		VII		VIII		IX		X	
	PDA	YPG	PDA	YPG	PDA	YPG	PDA	YPG	PDA	YPG	PDA	YPG	PDA	YPG	PDA	YPG	PDA	YPG	PDA	YPG
*A. tetracladia*	21.66	—	—	—	—	—	—	—	10.83	—	—	—	—	—	—	—	—	—	—	—
*A. pseudoglaucus*	3.33	—	—	—	—	3.33	—	—	—	—	—	—	—	—	—	—	—	—	1.83	—
*B. pseudobassiana*	— ^2^	—	—	—	—	—	—	—	40.33	—	—	—	—	—	—	—	—	0.67	—	—
*B. adusta*	—	—	3.33	—	8.33	—	6.67	5.33	11.67	29.33	—	1.50	—	7.50	5.17	0.67	7.33	0.05	11.67	5.20
*C. ramotenellum*	—	—	—	—	—	—	—	—	—	—	—	—	—	—	—	2.22	—	—	—	—
*H. mucida*	—	14.44	—	—	1.67	—	—	0.25	—	—	3.33	—	—	—	—	0.22	—	—	—	3.40
*Orbiliaceae* sp.	—	—	—	—	26.67	3.34	2.00	—	—	—	—	3.33	—	—	1.83	2.22	—	—	—	—
*P. album*	1.67	8.78	15.00	3.67	18.33	0.02	56.67	17.92	8.33	18.33	14.00	1.83	14.17	4.17	19.33	4.78	16.67	0.03	7.33	10.22
*P. hyalophaea*	58.33	0.44	11.87	21.66	3.33	8.36	10.33	4.67	6.67	11.00	6.67	2.17	7.17	8.67	—	6.44	—	3.05	—	5.17
*P. expansum*	6.67	—	38.33	—	—	—	—	—	—	—	—	—	—	—	—	—	—	—	—	—
*P. cumulodentata*	—	—	—	—	—	—	—	—	—	—	5.17	—	25.17	—	11.83	—	10.43	0.05	22.40	—
*P. hygrophilum*	—	2.22	—	21.00	—	—	—	—	—	5.00	—	—	—	4.33	—	—	—	0.03	—	8.33
Total species	5	4	4	3	5	4	4	4	5	4	4	4	3	4	4	6	3	6	4	5

**Table A2:** Fungi cultured from cryoconite holes (CFU × 10^2^ per 1 g) in the Werenskiold Glacier (Spitsbergen) at 24 °C on different media and incubation temperature: ^1^ I–X—study sites; ^2^ “—” means not detected.

Fungi	Study Sites																			
	I ^1^		II		III		IV		V		VI		VII		VIII		IX		X	
	PDA	YPG	PDA	YPG	PDA	YPG	PDA	YPG	PDA	YPG	PDA	YPG	PDA	YPG	PDA	YPG	PDA	YPG	PDA	YPG
*A. tetracladia*	— ^2^	—	0.01	—	—	—	—	—	—	—	—	—	—	—	—	—	—	—	—	—
*A. pseudoglaucus*	—	—	—	—	—	—	—	—	—	—	—	—	—	0.40	—	—	—	—	—	—
*A. sydowii*	—	—	—	—	—	—	—	—	—	—	—	—	—	—	—	0.33	—	—	—	—
*B. adusta*	—	—	—	—	—	—	—	—	—	—	—	—	—	—	0.15	—	—	0.84	2.00	—
*B. cinerea*	—	—	—	—	—	—	0.01	0.01	—	—	—	—	—	—	—	—	—	—	—	—
*Ch. globosum*	—	—	—	—	—	—	—	—	—	—	0.05	0.20	—	0.33	—	0.01	0.02	0.34	0.03	—
*C. allicinum*	—	1.83	—	0.04	—	0.01	—	0.2	—	0.34	—	0.11	—	—	—	—	—		—	0.005
*C. cladosporioides*	—	—	—	—	—	0.33	—	—	—	—	0.07	—	0.51	—	—	—	—	0.01	—	—
*C. ramotenellum*	—	—	—	—	—	—	—	—	1.25	—	0.01	0.10	—	—	—	—	—	—	—	—
*Dothideomycetes* sp.	—	—	—	—	—	—	—	—	—	—	—	—	—	—	0.01	—	—	—	—	—
*F. rabenhorstii*	—	—	0.03	—	—	—	—	—	0.01	—	0.04	—	—	—	—	—	—	—	—	—
*H. mucida*	8.33	—	0.02	—	0.01	—	—	—	—	—	—	—	—	—	—	—	—	—	—	—
*I. pannonica*	—	—	0.05	—	—	—	0.04	—	0.01	—	0.03	—	—	—	—	—	0.02	—	0.03	—
*Orbiliaceae* sp.	0.05	—	0.15	0.06	0.03	—	—	—	2.60	—	0.22	—	1.54	—	0.02	—	0.09	—	0.14	0.005
*P. hyalophaea*	—	0.01	—	—	0.02	—	—	—	—	0.02	—	—	—	—	—	—	—	—	—	0.015
*P. expansum*	0.03	—	—	—	0.22	—	0.02	—	—	—	0.02	—	0.02	—	0.05	—	—	—	1.00	—
*P. sumatraense*	—	—	—	0.01	—	—	—	—	—	—	—	—	—	—	—	—	—	—	—	1.00
*P. velutinum*	—	—	—	0.11	—	—	—	—	—	—	—	—	0.90	—	—	—	—	—	0.01	—
*P. cumulodentata*	—	—	—	—	—	—	—	—	—	—	0.90	—	—	—	—	—	4.11	—	—	—
*P. hygrophilum*	—	—	—	—	—	0.37	—	—	—	—	—	—	—	—	—	0.02	—	—	—	—
*T. versicolor*	—	—	—	—	—	—	—	—	—	—	—	—	—	—	—	—	1.11	—	—	—
Total species	3	2	4	4	4	3	3	2	4	2	7	3	4	2	4	3	5	3	6	4
